# High-level tolerance to triclosan may play a role in *Pseudomonas aeruginosa *antibiotic resistance in immunocompromised hosts: evidence from outbreak investigation

**DOI:** 10.1186/1756-0500-5-43

**Published:** 2012-01-19

**Authors:** Silvia D'Arezzo, Simone Lanini, Vincenzo Puro, Giuseppe Ippolito, Paolo Visca

**Affiliations:** 1 National Institute for Infectious Diseases "Lazzaro Spallanzani", I.R.C.C.S., Rome, Italy; 2 Department of Biology, University "Roma Tre", Rome, Italy; 3 Dipartimento di Epidemiologia e Ricerca pre-clinica, Istituto Nazionale per le Malattie Infettive, Via Portuense 292, 00149 Roma, Italia

## Abstract

**Background and methods:**

*Pseudomonas aeruginosa *is a major infectious threat to immunocompromised patients. We recently reported a fatal epidemic of multidrug-resistant *P. aeruginosa *in an onchoematology unit, linked to massive contamination of a triclosan-based disinfectant. The aim of this study is to evaluate the antimicrobial activity of triclosan and chlorhexidine digluconate against the epidemic strain of *P. aeruginosa*, to confirm the hypothesis that the soap dispenser acted as a continuous source of the infection during the outbreak, and to explore the potential role of triclosan in increasing the level of resistance to selected antibiotics.

Susceptibility tests and time-kill assays for disinfectans were performed using two commercial formulations containing triclosan and chlorhexidine digluconate, respectively. Antibiotic susceptibility testing was performed by the broth microdilution method.

**Findings:**

The *P. aeruginosa *epidemic strain exhibited an extremely high level of triclosan resistance (apparent MIC = 2,125 mg/L), while it was markedly susceptible to chlorhexidine digluconate (apparent MIC = 12.5 mg/L). Upon gradual adaptation to triclosan, the epidemic strain survived for a long period (> 120 h) in the presence of 3,400 mg/L (equivalent to 1.6 × MIC) of triclosan, concomitantly increasing the resistance to six antibiotics that are typical substrates of drug efflux pumps of the resistance nodulation division family. This effect was reversed by efflux pump inhibitors.

**Conclusions:**

The epidemic *P. aeruginosa *strain was resistant to triclosan and its previous exposure to triclosan increases antibiotic resistance, likely through active efflux mechanisms. Since *P. aeruginosa *can become tolerant to elevated triclosan concentrations, the use of triclosan-based disinfectants should be avoided in those healthcare settings hosting patients at high risk for *P. aeruginosa *infection.

## Background

The Gram-negative, rod shaped, aerobic bacterium *Pseudomonas aeruginosa *is a leading cause of severe infections in hospitalized subjects [1]. Colonization of the hospital environment, which may also involve antiseptic solutions, is a well-established mechanisms of *P. aeruginosa *persistence and spread in healthcare setting [2,3].

We recently described an outbreak of multidrug-resistant (MDR) *P. aeruginosa *infection which occurred in a oncohematology unit in Italy [3]. The outbreak involved 5 patients, 3 of whom died. Isolates from all 5 cases of infection showed an identical antibiotic resistance pattern (i.e. resistant to fluoroquinolones and aminoglycosides and susceptible to beta-lactams), all belonged to sequence type 175, and showed identical pulsotype and RAPD type, forming a single epidemiological cluster. We were also able to trace back the source of infection to a common soap dispenser which was found to be heavily contaminated with the same *P. aeruginosa *isolates infecting the cases. This soap dispenser used to be manually refilled with commercial preparations of either 4% clorexidine digluconate or 0.5% triclosan in 3 months rotation; at the time infections set in, the dispenser contained the triclosan soap.

Triclosan is an antiseptics commonly used in hospitals because of the broad antimicrobial activity [4]. However, concern has been raised over triclosan efficacy since some bacterial species are endowed with intrinsic resistance to this compound [4]. *P. aeruginosa *is resistant to triclosan as the consequence of the expression of multiple mechanisms, including active efflux from the cells mediated by the resistance nodulation division (RND) family of efflux pumps [5], the presence of a triclosan-resistant enoyl-acyl carrier protein reductase (FabV) [6], outer membrane impermeability, and the expression of a triclosan-specific pump (TriABC-OpmH) [7,8]. Some of these mechanisms are also associated with the development of resistance to some clinically significant antibiotics, raising the additional concern that, under certain circumstances, inappropriate use of triclosan-containing disinfectants may select for resistance against clinically useful drugs [9,10]. The aim of this study was to evaluate the activity of two commercial disinfectants containing triclosan and chlorhexidine digluconate against the epidemic *P. aeruginosa *strain, in order to confirm the hypothesis that the soap dispenser containing triclosan may have actually acted as a continuous source of the infection during the outbreak [3], and to explore the potential role played by triclosan in enhancing the level of resistance to selected antibiotics.

## Methods

### Bacterial isolates

Six different *P. aeruginosa *strains were isolated throughout the outbreak investigation, as previously reported [3]. *P. aeruginosa *strain L2 was isolated in the triclosan-containing soap dispenser and isolates L3 and L4 from water outlets in patients rooms (environmental isolates). *P. aeruginosa *isolates 10, 11 and 13 were isolated from patients' biological specimens (clinical isolates). Isolates L2, 10, 11 and 13 all belonged to sequence type 175 and were indistinguishable by pulsotyping, RAPD and MLST analysis, while isolates L3 and L4 showed a distinct type [3]. After primary isolation, isolates were sub-cultured for 4-6 times in Mueller-Hinton (MH, Oxoid, Milan, Italy) agar, and then stored frozen in MH broth.

### Disinfectants

Two commercial disinfectants were used in this study, designated *product A *and *product B*, whose composition (% w/v in water) is: (*A*) 0.5% 2,4,4'-trichloro-2'-hydroxy-diphenyl-ether (triclosan), 10% sodium lauryl-myristyl ether sulfate, 3% coconut glycerol, 2.5%coconut amido propyl betaine, and 1.5% alcohol ethoxylate; (*B*) 4% chlorhexidine gluconate, 15% polysorbate 20, 5% coconut diethanolamide, 4% alkyl polyglucoside, 1% polyethylene glycol (PEG)-150, 1% isopropyl alcohol, 0.1% essence, and 0.005% E 124.

### Susceptibility tests for disinfectants

Taking into account the very poor solubility of triclosan in aqueous solutions [11], the antimicrobial susceptibility tests and the time kill assay were performed using the commercial soap formulations. In the commercial *product A*, triclosan is well soluble (it is 5,000 mg/L), and its addition to MH broth (that was adequately concentrated to have a final concentration of 1 × MH broth in all assays) did not result in any precipitation of the disinfectant.

*P. aeruginosa *was grown to the early stationary phase in MH broth. Bacterial cultures were diluted to 0.5 MacFarland turbidity in MH broth (~5 × 10^8 ^CFU/ml). This suspension was diluted 1:10 (~5 × 10^7 ^CF/mL) in MH broth, and 10 μl were used as inoculum in 96-well microtiter plates (Costar, Cambridge, Massachusetts) containing 190 μl of serial two-fold dilutions of the disinfectant soaps in 1 × MH broth. To test the highest concentration of triclosan (4,250 mg/L, which corresponds to the minimum soap dilution), 20 μl of 10 × concentrated MH broth were mixed with 170 μl of undiluted triclosan soap (*product A*) containing 5,000 mg/L triclosan and 10 μl of bacterial inoculum (~2.5 × 10^6^). All other dilutions were prepared by combining appropriate volumes of 10 × concentrated MH broth, triclosan soap, sterile water, and the standard 10-μl bacterial inoculum.

To test the susceptibility of *P. aeruginosa *to chlorexidine digluconate (*product B*), standard two-fold dilutions of the disinfectant were generated in 1 × MH broth and inoculated with ~2.5 × 10^6 ^CFU/mL in 96-well microtiter plates (Costar).

Since the disinfectant soaps were commercial mixtures of triclosan or chlorhexidine with other ingredients which may or may not be inert, the observed minimal inhibitory concentrations (MICs) were called "apparent MICs" (a-MICs). The a-MIC was expressed as the minimal concentration of the disinfectant in the commercial formulation causing no growth after 48-h incubation at 37°C [12,13], with chlorhexidine > 50 mg/L [14] and triclosan > 128 mg/L [13] as resistance breakpoints. Each experiment was performed in triplicate.

### Determination of the bactericidal activity for disinfectants

*P. aeruginosa *L2 suspensions (~5 × 10^7 ^CFU/mL) were made in MH broth supplemented with either *product A *(triclosan range: 1,062-4,250 mg/L) or *product B *(chlorhexidine digluconate range: 12.5-1,250 mg/L), and incubated at 37°C. The two products were diluted in MH broth, prepared at the appropriate concentration in order to compensate for the addition of an appropriate volume of disinfectant and a constant bacterial inoculum. At defined intervals, aliquots were neutralized by rapid serial dilution in MH broth supplemented with 0.5% lecithin and 4% polysorbate 20 [15], plated on MH agar plates, and examined after 24-h incubation at 37°C for determination of viable counts.

### Adaptation to high triclosan concentration

The frozen stock of *P. aeruginosa *L2 was readapted to triclosan by passages in gradually increasing triclosan concentrations. Inocula of ~5 × 10^5 ^CFU/mL were serially cultured in stepwise increasing triclosan concentrations in MH broth at 37°C, starting with 1/2 the MIC and doubling the concentration at each passage until evidence of full growth.

### Antimicrobial susceptibility testing

The susceptibility of triclosan-adapted and unadapted *P. aeruginosa *to a panel of antibiotics which are typically exported by RND efflux pumps [16], namely tetracycline, ciprofloxacin, amikacin, levofloxacin, carbenicillin and chloramphenicol (all from Sigma-Aldrich) was determined by the broth microdilution method according to the CLSI guidelines [12] either with or without the RND efflux pump inhibitor phenyl-arginine-β-naphthylamide (PAβN) or the protonophore carbonyl cyanide m-chlorophenylhydrazone (CCCP) (Sigma-Aldrich) at the predetermined concentrations of 50 μM and 100 μM, respectively. Each experiment was performed in triplicate.

## Results

### Susceptibility to triclosan and chlorhexidine digluconate

Elevated triclosan resistance (a-MIC = 2,125 mg/L for isolates 10, 11, 13 and L2; a-MIC = 4,250 mg/L for isolates L3 and L4) was observed for both clinical and environmental *P. aeruginosa *isolates tested after primary isolation and serial passages in MH broth. Conversely, all isolates were markedly susceptible to chlorhexidine digluconate (a-MIC = 12.5 mg/L).

### Determination of the bactericidal activity for disinfectants

Time-kill kinetics of the two disinfectants were determined for the *P. aeruginosa *L2 soap isolate, regarded to as the prototypic epidemic strain.

**Figure 1:**
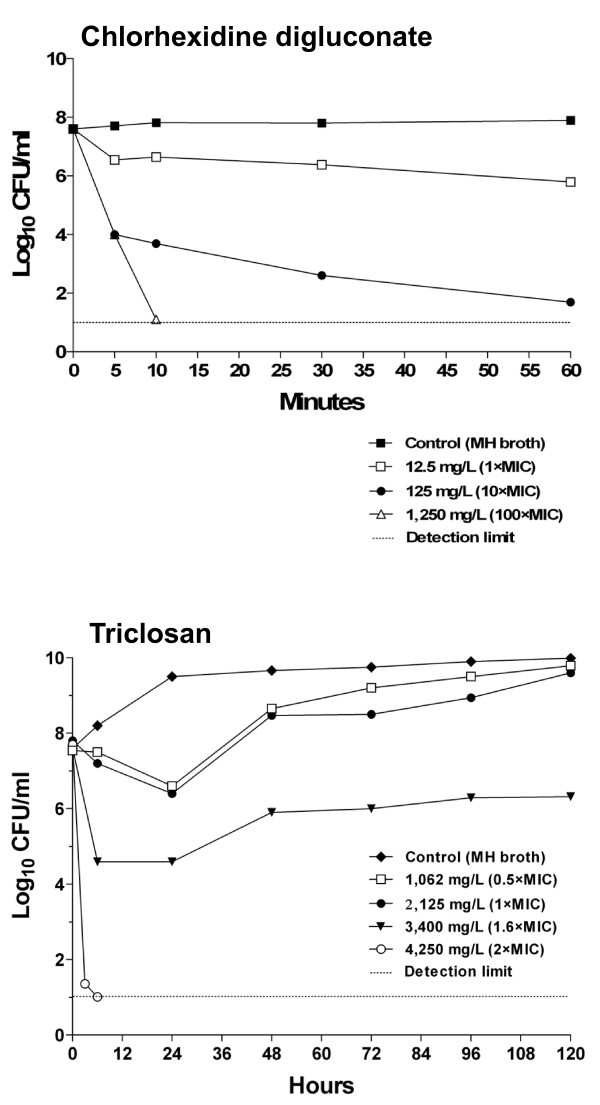
**Time-kill kinetics of *P. aeruginosa *L2 in MH broth supplemented with chlorhexidine digluconate (*product B*, upper panel) or triclosan (*product A*, lower panel) at given concentrations**.

Chlorhexidine digluconate at ≥ 125 mg/L exerted a rapid bactericidal effect (> 3 log_10 _reduction of viable counts) within 5 min of contact with *P. aeruginosa *L2 cells. This concentration is far below the actual concentration, 40,000 mg/L, provided by the manufacturer in the commercial *product B *(Figure 1, upper panel).

The triclosan-containing *product A *was much less effective in killing *P. aeruginosa *L2 cells (Figure 1, lower panel). Only the highest triclosan concentration achievable in MH broth (4,250 mg/L, equivalent to 2 × MIC) exerted strong bactericidal activity within 3 h of contact with *P. aeruginosa *cells (> 6 log_10 _reduction of CFU/ml). When exposed to triclosan concentrations of 1,6 × MIC (3,400 mg/L), L2 cells were killed during the initial 6 h (> 3 log_10 _reduction of CFU/ml), but the residual population could then survive for a long period (> 120 h) in the presence of this concentration of the disinfectant. At triclosan concentration of 2,125 and 1,062 mg/L (equivalent to 1× and 0,5 × MIC, respectively) ~1 log_10 _reduction of cell viability was observed relative to the inoculum size at 24 h, but afterwards growth was restored and the bacterial population reached the same density as the control in triclosan-free medium after 120 h.

### Adaptation to triclosan results in increased levels of antibiotic resistance

In *P. aeruginosa*, triclosan resistance is mediated at least by 4 efflux pumps, belonging to the RND family (MexAB-OprM, MexCD-OprJ, MexEF-OprN, and MexXY-OprM), which can also drive active extrusion of various antibiotics [5,10]. Therefore, we tested the susceptibility to six antibiotics (tetracycline, ciprofloxacin, amikacin, levofloxacin carbenicillin and chloramphenicol) typically exported via RND pumps in unadapted and triclosan-adapted *P. aeruginosa *L2 cells (Table 1). We observed that the efflux pump inhibitors PAβN or CCCP, at concentrations which do not affect bacterial growth, decreased the MIC of tetracycline, ciprofloxacin, amikacin, levofloxacin, carbenicillin and chloramphenicol by a factor 2-4 in triclosan-unadapted *P. aeruginosa *cells. Remarkably, adaptation of *P. aeruginosa *L2 to sub-lethal concentrations of triclosan (up to 3,400 mg/L, see Methods and Table 1) resulted in a 2-fold increase of MICs for all six antibiotics. Efflux pump inhibitors (PAβN or CCCP) restored antibiotic susceptibility of the triclosan-adapted isolate to the same or even higher level than the unadapted isolate (2-4 fold reduction of MICs, Table 1), suggesting that active antibiotic efflux was enhanced in *P. aeruginosa *cells following triclosan exposure.

**Table 1:** Antibiotic susceptibility in unadapted and triclosan-adapted *P.aeruginosa*

*P. aeruginosa *L2	Antibiotic MIC (mg/L) ^b^					
	
	TET	CIP	AMK	LVX	CAR	CHL
Unadapted	32	16	4	8	128	128
Unadapted plus PAβN (50 μM)	16	4	2	2	64	64
Unadapted plus CCCP (100 μM)	16	8	2	4	64	64
Adapted ^a ^to 3,400 mg/L triclosan	64	32	8	16	256	256
Adapted ^a ^to 3,400 mg/L triclosan plus PAβN (50 μM)	32	8	4	4	64	64
Adapted ^a ^to 3,400 mg/L triclosan plus CCCP (100 μM)	32	16	4	8	64	64

^a^Adaptation was achieved by serial subculturing in stepwise increasing triclosan concentrations up to 3,400 mg/L triclosan (see Methods). The experimental data were confirmed by three independent replicates

^b^Breakpoint criteria were as follows. TET: susceptible, ≤ 4 mg/L; intermediate, 8 mg/L; resistant, ≥ 16 mg/L; CIP: susceptible, ≤ 1 mg/L; intermediate, 2 mg/L; resistant, ≥ 4 mg/L; AMK: susceptible, ≤ 16 mg/L; resistant, ≥ 32 mg/L; LVX: susceptible, ≤ 2 mg/L; intermediate, 4 mg/L; resistant, ≥ 8 mg/L; CAR: susceptible, ≤ 128 mg/L; intermediate, 256 mg/L; resistant, ≥ 512 mg/L; CHL: susceptible, ≤ 8 mg/L; intermediate, 16 mg/L; resistant, ≥ 32 mg/L [12]

*MIC *minimal inhibitory concentration; *PAβN *RND pump inhibitor phenyl-arginine-β-naphthylamide; *CCCP *protonophore carbonyl cyanide m-chlorophenylhydrazone; *TET *tetracycline; *CIP *ciprofloxacin; *AMK *amikacin; *LVX *levofloxacin; *CAR *carbenicillin; *CHL *chloramphenicol

## Discussion

A *P. aeruginosa *strain, which eventually caused a fatal epidemic cluster, was able to contaminate and likely replicate within a triclosan-soap dispenser. Triclosan is an anionic, lipophilic compound that is very poorly soluble in water [11]. However, previous researchers have reported that various solubilizers, such as the surfactant sodium lauryl sulphate, increase the solubility of triclosan from 80- to 6,000-fold. Micellar solubilization and the formation of either salts or complexes are postulated as possible mechanisms for the increase in the solubility of triclosan [11]. In this study, we decided to perform the antimicrobial susceptibility tests and the time kill assays using the commercial soap *product A*, in which triclosan (5,000 mg/L) is kept in solution by 10% sodium lauryl-myristyl ether sulfate. Assuming that this surfactant, or any other additive of *product A*, may have increased the effectiveness of triclosan in killing *P. aeruginosa*, this would paradoxically imply that the actual *P. aeruginosa *resistance to triclosan alone would be higher than that observed in our experimental system.

Determination of the bactericidal activity for triclosan confirmed that the epidemic strain could have contaminated the soap dispenser most probably because of its remarkable tolerance to triclosan. In fact this epidemic strain was originally isolated at relatively high concentration (5 × 10^4 ^CFU/mL) from the dispenser containing the triclosan-based commercial soap (*product A*) for healthcare workers' hand washing [3]. These findings provide additional biological evidence to confirm our previous hypothesis, based on molecular identity between patients' and soap *P. aeruginosa *isolates [3], that the contaminated soap dispenser may have acted as the primary source of infection. We can also suppose that the activity of triclosan in the commercial formulation was reduced by partial inactivation of triclosan by micellar entrapping on inclusion which may have reduced the amount of free triclosan available in solution.

Conversely, the chlorhexidine-based disinfectant, which was employed in rotation of 3 months with triclosan in the oncohematology unit [3], had strong and fast bactericidal effect at concentrations far below those recommended by the manufacturer for practical use.

Another interesting result was that the level of resistance to some antibiotics, namely tetracycline, fluoroquinolones, aminoglycosides, carbenicillin and chloramphenicol, was increased by previous adaptation of *P. aeruginosa *to triclosan. As reported in other studies [10,16], triclosan may induce the expression of multidrug efflux systems belonging to RND family in *P. aeruginosa*. These systems are relatively nonspecific in substrate recognition and enables bacteria to pump out numerous chemically unrelated substances, such as antibiotics and biocides. Here, we report that the pattern of increasing resistance to antibiotics was likely to be driven by activation of efflux pumps belonging to the RND family; in fact, the RND efflux pump inhibitor PAβN and the protonophore CCCP restored the baseline antibiotic susceptibility in the triclosan-adapted strain. Thus, while the link between triclosan usage and development of clinically significant clinical resistance to antibiotics remains controversial [9,10], here we provide evidence that previous exposure to triclosan elevates the level of antibiotic resistance. Although the differences in MICs obtained for the different antibiotics are small (2-fold), they provide a clear evidence of the overall trend towards increasing antibiotic resistance associated with adaptation to tricosan. Increased resistance was also observed for those antibiotics to which *P. aeruginosa *L2 cells showed elevated baseline resistance. As noted for carbenicillin, adaptation to triclosan converted the susceptible phenotype (MIC ≤ 128 mg/L) into intermediate resistant (MIC = 256 mg/L).

To the best of our knowledge, this is the first time that such a high tolerance to triclosan is documented in a clinical *P. aeruginosa *isolate, since previous studies on both uncharacterized and type (PAO1) *P. aeruginosa *strains reported actual MICs of ~ 1,000 mg/L [17].

Some issues limit the generalizability our findings. Firstly, our data come from a single epidemic event, and therefore they may not apply in the same way to seemingly similar episodes in other institutions or to general clinical practices as biological features of the outbreak strain (e.g. virulence, infectivity, and/or baseline resistance patterns) and/or local situation (e.g. infection control policies, mode of transmission, and/or risk factors) may be unique. Secondly, we could not quantify the actual triclosan concentration in the triclosan-based commercial soap formulation *- *as it was in the soap dispenser at the time of the outbreak - since the oncohematology unit was closed due to legal issue, and the only soap sample obtained was insufficient for chemical analysis. Therefore we cannot rule out that accidental dilution (below the commercial concentration) of triclosan soap due to refilling procedure might have played a preeminent role in facilitating the growth of *P. aeruginosa *in the dispenser and the eventual spreading of the infection. Finally, we only performed phenotypic assays, and we cannot provide a genetic basis for the remarkably high resistance to triclosan of the *P. aeruginosa *outbreak strain. Intrinsic outer membrane impermeability, resistance of the FabV enoyl-acyl carrier protein reductase, expression of an additional triclosan-specific pump (TriABC-OpmH) or other unknown mechanisms could have acted synergistically with efflux in determining the unprecedented triclosan resistance observed our isolates.

Despite these limitations, this study provide biological evidence to confirm that an unprecedented environmental source, which was identified as a triclosan soap dispenser, actually acted as a continuous source of infection during the outbreak. In addition, we provide hints that exposure to sub-lethal concentration of triclosan may contribute to antibiotic resistance in *P. aeruginosa *clinical isolates.

## Conclusions

Since certain strains of *P. aeruginosa *can grow in the presence of triclosan concentrations similar to those present in commercial soap formulations, triclosan-based disinfectants can incidentally be contaminated and act as a continuous source of *P. aeruginosa *in healthcare settings. The concentration of triclosan-based disinfectants meant to be used in those facilities admitting patients at high risk for *P. aeruginosa *infection should be reassessed.

## Abbreviations

*P. aeruginosa*: *Pseudomonas aeruginosa*; RND: resistance nodulation and cell division; w/v: weight/volume (as concentration measure); CFU: colony forming unit; MIC: minimal inhibitory concentration; PAβN: pump inhibitor phenyl-arginine-β-naphthylamide; CCCP: pump inhibitor protonophore carbonyl cyanide m-chlorophenylhydrazone; MH: Mueller-Hinton; μM: micro molar; mg: milligrams; mL: millilitre; °C: Celsius degree; L: litre.

## Competing interests

The authors declare that they have no competing interests.

## Authors' contributions

SDA carried out the phenotypic assays and drafted the manuscript. SL and VP participated in the design of the study and obtained samples during outbreak investigations. VP and GI conceived of the study, and participated in its design and coordination and helped to draft the manuscript. All authors read and approved the final manuscript.

## Funding

This work was supported by a grant from Ministero della Salute- Ricerca Corrente 2008.
